# The Small-Molecule Inhibitor MRIA9 Reveals Novel Insights into the Cell Cycle Roles of SIK2 in Ovarian Cancer Cells

**DOI:** 10.3390/cancers13153658

**Published:** 2021-07-21

**Authors:** Monika Raab, Marcel Rak, Roberta Tesch, Khayal Gasimli, Sven Becker, Stefan Knapp, Klaus Strebhardt, Mourad Sanhaji

**Affiliations:** 1 Department of Gynecology, Medical School, Goethe University, 60590 Frankfurt, Germany; monika.raab@kgu.de (M.R.); khayal.gasimli@kgu.de (K.G.); sven.becker@kgu.de (S.B.); strebhardt@em.uni-frankfurt.de (K.S.); 2 Institute of Pharmaceutical Chemistry, Johann Wolfgang Goethe University, Max-von-Laue-Str. 9, 60438 Frankfurt, Germany; rak@pharmchem.uni-frankfurt.de (M.R.); tesch@pharmchem.uni-frankfurt.de (R.T.); knapp@pharmchem.uni-frankfurt.de (S.K.); 3 Structural Genomics Consortium (SGC), Buchmann Institute for Life Sciences, Johann Wolfgang Goethe University, Max-von-Laue-Str. 15, 60438 Frankfurt, Germany; 4 German Translational Cancer Network (DKTK), Frankfurt Cancer Institute (FCI), 60438 Frankfurt, Germany; 5 German Cancer Consortium (DKTK)/German Cancer Research Center, 69120 Heidelberg, Germany

**Keywords:** Salt inducible kinase 2 (SIK2), the small molecule inhibitor MRIA9, spindle mispositioning, chromosomal instability, ovarian cancer, paclitaxel sensitization

## Abstract

**Simple Summary:**

The current standard therapy of ovarian cancers comprises a reductive surgery followed by a combination of taxane-platinum-based primary chemotherapy. However, despite an initial positive response, patients in the advanced stage showed relapse within months or even weeks. Thus, there is a need to find combinatorial therapies that permit overcoming the paclitaxel-associated resistance in patients. Here, we found that MRIA9, a newly developed small-molecule inhibitor of the salt-inducible-kinase 2, interferes with the cell division of cancer cells. More importantly, MRIA9 increases paclitaxel efficiency in eliminating ovarian cancer cells and patient derived cancer cells by inducing apoptosis or programmed cell death. Thus, our study indicates that MRIA9 might represent a novel therapeutical tool for translational studies to overcome paclitaxel resistance in ovarian cancer.

**Abstract:**

The activity of the Salt inducible kinase 2 (SIK2), a member of the AMP-activated protein kinase (AMPK)-related kinase family, has been linked to several biological processes that maintain cellular and energetic homeostasis. SIK2 is overexpressed in several cancers, including ovarian cancer, where it promotes the proliferation of metastases. Furthermore, as a centrosome kinase, SIK2 has been shown to regulate the G2/M transition, and its depletion sensitizes ovarian cancer to paclitaxel-based chemotherapy. Here, we report the consequences of SIK2 inhibition on mitosis and synergies with paclitaxel in ovarian cancer using a novel and selective inhibitor, MRIA9. We show that MRIA9-induced inhibition of SIK2 blocks the centrosome disjunction, impairs the centrosome alignment, and causes spindle mispositioning during mitosis. Furthermore, the inhibition of SIK2 using MRIA9 increases chromosomal instability, revealing the role of SIK2 in maintaining genomic stability. Finally, MRIA9 treatment enhances the sensitivity to paclitaxel in 3D-spheroids derived from ovarian cancer cell lines and ovarian cancer patients. Our study suggests selective targeting of SIK2 in ovarian cancer as a therapeutic strategy for overcoming paclitaxel resistance.

## 1. Introduction

Salt inducible kinase (SIK) was first isolated from the adrenal glands of high salt diet-fed rats in 1999 [1]. This family of serine/threonine kinases encompasses 3 members, SIK1–3, all functioning as metabolic transmitters and mediators of energy homeostasis and belonging to the AMP-activated protein kinase (AMPK) family [2,3]. The members of the SIK family show different expression patterns indicating tissue-specific functions. While SIK1 is highly expressed in the adrenal cortical gland and in adipose and neural tissues, the expression of SIK2 and SIK3 is ubiquitous in human organs with specific enrichment in the adipose and neural tissues, respectively [2,4,5]. As an isoform of the SIK family, SIK2 maintains cell homeostasis during nutrient deprivation by controlling the cAMP element-binding protein (CREB)-dependent gene expression [6]. SIK2 reduces the glucose uptake in muscle cells and adipocytes, and through phosphorylation of the histone acetyltransferase p300, it decreases lipogenesis and ketogenesis [7]. SIK2 is also involved in several signaling pathways, including the PI3K-AKT-mTOR pathway, the Hippo-Yap pathway, the LKB1-HDAC, and the cAMP-PKA axis [4]. SIK kinases are highly expressed in several cancer types. Remarkably, high expression levels of SIK2 in ovarian cancer suggest a role in tumor progression [4,8,9,10,11]. Notably, a recent study reported that SIK2 is overexpressed in ovarian cancer metastasis in the omentum, an adipocyte-rich abdominal cavity representing the predilection site for ovarian metastasis. The activation of SIK2 by the omental adipocytes promotes the proliferation and the growth of ovarian cancer metastases [9]. Furthermore, SIK2 has been identified as a centrosome kinase regulating the G2-M transition, and its depletion or inhibition sensitizes ovarian cancer to paclitaxel-based chemotherapy [10,12]. All these findings argue for SIK2 as a promising target in suppressing metastatic ovarian cancer. Although several SIK inhibitors have already been investigated [7,10,13,14], all these reagents exhibit a common problem: the lack of specificity and significant off-target activity limiting their use as mechanistic tools for studying the role of SIK kinases in cancer and other diseases [15]. Hence, there has been a need to optimize small molecules to achieve better inhibitor selectivity. Recently, we addressed this issue by developing a small molecule with potent panSIK activity [15]. Kinome-wide selectivity screening and cellular on-target assays identified only the p21-activated kinase family members 1–3 (PAK1-3) as off-targets of the lead compound MRIA9. We used this new chemical tool and showed that MRIA9 strongly impeded centrosome function, caused mitotic spindle mispositioning in ovarian cancer cell lines, and sensitized ovarian cancer cells and patient derived 3D-spheroids to paclitaxel treatment.

## 2. Results

### 2.1. MRIA9 Inhibits SIK2 Catalytic Activity in Ovarian Cancer Cells

In recent years, the role of kinases of the SIK family in regulating cellular metabolism has been well established [16,17]. Intriguingly, recent data added a novel and unexpected role to the SIK2 isoform in regulating the cell cycle. Several studies have demonstrated that SIK2 is a centrosome kinase involved in mitosis regulation by facilitating centrosome separation during the G2 phase, and preclinical studies have reported the impact of SIK2 depletion in boosting the sensitivity of ovarian cancer cells to paclitaxel treatment [9,10,12]. However, most cited studies adopted an RNAi-based approach to decipher the implication of SIK2 loss of function yet transposing this technology into a clinical benefit for patients has remained challenging. Hence, there is an unmet need for small molecule inhibitors that selectively target SIK in cancer cells.

Recently, we developed a potent pan-SIK inhibitor, MRIA9, which displayed high selectivity against SIK kinases [15]. MRIA9 is an ATP-competitive inhibitor with an in vitro IC_50_ of 180 nM determined by nanoBRET (Bioluminescence Resonance Energy Transfer) assays for the kinase isoform SIK2. Moreover, at a concentration of 1 µM, MRIA9 showed strong SIK2 selectivity against 443 kinases in radiometric enzyme kinetic assay (PanQinase^®^) and evaluation of all potential off-targets in cell-based on-target assays identified only PAK1-3 as off-targets suggesting that MRIA9 can be used as a chemical probe to investigate SIK function in cellular systems [15].

**Figure 1:**
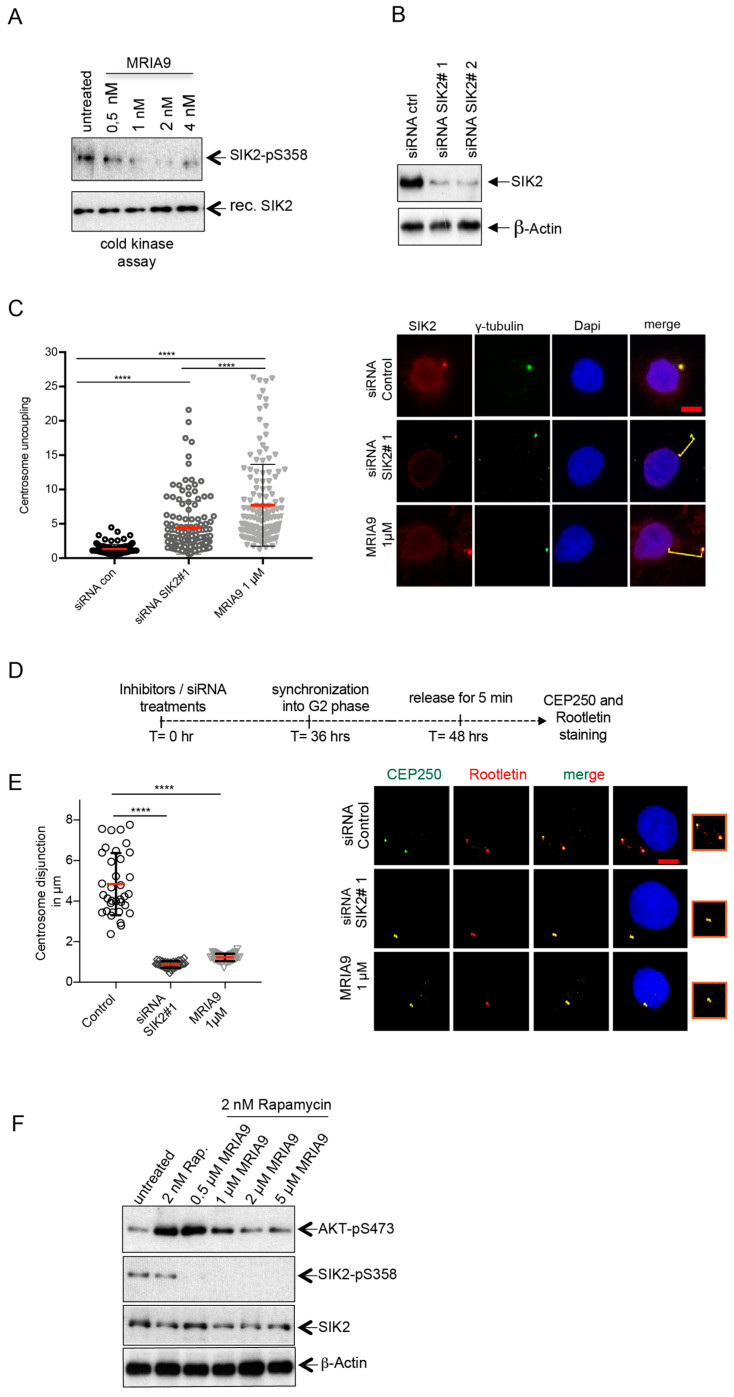
MRIA9 potently inhibits SIK2 and prevents the centrosome disjunction during the late G2 phase. (**A**) Recombinant GST-SIK2 was subjected to a cold kinase assay in the presence of increasing concentrations of MRIA9 (0.5 to 4 nM). The reaction was immunoblotted for SIK2-pS385 to monitor the autophosphorylation and for SIK2 using specific antibodies. (**B**) SKOV-3 cells were treated for 48 h with two siRNA against SIK2. Cell lysates were analyzed by immunoblotting for SIK2 and β-Actin. (**C**) MRIA9 causes nuclear centrosome uncoupling (NCU). SKOV-3 cells were treated with siRNA SIK2#1 and 1 µM MRIA9 for 48 h. Cells were fixed and processed for immunofluorescence using gamma-tubulin, SIK2, and DAPI. (**left**) The centrosome nuclear distances were measured in µm and plotted as a scatter plot with mean ± SD. Values were calculated from at least 100 cells per treatment and statistically analyzed (**** *p* < 0.001). (**Right**) Representative figures of NCU in the different treatment groups. The yellow lines indicate the centrosome nuclear distance in µm. Scale bar = 10 µm. (**D**) Scheme of the experimental procedure. SKOV-3 cells were treated with siRNA SIK2#1 and 1 µM MRIA9 and synchronized into the G2 phase. Cells were shortly released into mitosis, then fixed and processed for immunofluorescence using CEP250, Rootletin, and DAPI. (**E**) MRIA9-induced inhibition of SIK2 results in centrosome disjunction failure. (**Left**) The centrosome separation in the different treatments was assessed by measuring the distance in µm between the separated CEP250 and Rootletin signals. The values were represented as a scatter plot. Values were calculated from at least 35 cells per treatment and statistically analyzed (**** *p* < 0.001). (**Right**) Representative figures of centrosome disjunction failures in the different treatment groups. Insets: Magnifications showing the centrosome disjunction in the different treatment groups. Scale bar = 10 µm. (**F**) SKOV-3 cells were first treated with 2 nM rapamycin for 16 h and subsequently treated with increasing concentration of MRIA9 (0.5 to 5 µM) up to 48 hrs. Cells were lysed and immunoblotted for AKT-pS473, SIK2-p378, SIK2, and β-Actin using specific antibodies.

We were interested to see whether MRIA9 would also abrogate SIK2 auto-activation by phosphorylation. We addressed this using recombinant GST-SIK2 fusion protein, which was incubated with increasing concentrations of MRIA9 *in vitro*, and we monitored the SIK2 auto-phosphorylation at Ser385, a marker of SIK2 catalytic activity [9], using a specific phospho-antibody (Figure 1A). SIK2 autophosphorylation diminished in a concentration-dependent manner, indicating the efficiency of MRIA9 in inhibiting SIK2 kinase activation at low nM concentrations (Figure 1A). Further, using immunofluorescence (IF) staining of SIK2 and γ-tubulin, we measured the nucleus-centrosome distance in SKOV-3 cells treated with siRNA SIK2#1 (Figure 1B) or 1 µM MRIA9. This experiment demonstrated that depletion or the MRIA9-dependent inhibition of SIK2 resulted in nuclear-centrosome uncoupling (NCU) (Figure 1C), which confirmed the results of prior studies [10,12]. However, the most substantial increase in NCU distance was measured after inhibiting SIK2 with 1 µM MRIA9 compared with control cells or with SIK2 depleted cells (from 1.324 µm to 4.434 µm for the siRNA control vs. siRNA SIK2#1 and from 4.434 µm to 7.703 µm for siRNA SIK2 vs. MRIA9 1 µM) (Figure 1C).

The role of SIK2 in triggering the centrosome disjunction during the G2 phase has already been reported [10,12]. Through phosphorylation of the centrosomal protein CEP250 at Ser2392, SIK2 facilitates the dissociation of the centrosome cohesion, thus promoting centrosome separation and the assembly of the mitotic spindle [10,12]. To investigate whether MRIA9-induced inhibition of SIK2 interferes with the centrosome disjunction during the late G2 phase, we treated SKOV-3 cells with siRNA SIK2#1 or with 1 µM MRIA9 for 36 h and synchronized the cells using the CDK1 inhibitor RO3306 (5 µM) for 16 h [18]. The cells were released into the early mitotic phases, fixed, and stained with CEP250, Rootletin for IF analysis (Figure 1D). Both the knockdown as well as MRIA9-induced inhibition of SIK2 prevented the centrosome disjunction during late G2 (Figure 1E), corroborating previous findings and suggesting the specificity and efficiency of MRIA9 in inhibiting the catalytic activity of SIK2.

It has been described that SIK2 kinase modulates the PI3K/AKT pathway in metastatic ovarian cancer [9]. Through direct phosphorylation of PI3K-S154 within the p85-α regulatory subunit, SIK2 significantly increases the in vitro activity of PI3K and the phosphorylation of its downstream target, the AKT kinase on Ser473 [9,19]. Based on these reports, we asked whether MRIA9-dependent inhibition of SIK2 can reduce PI3K/AKT kinase activity by monitoring the status of AKT-pS473. In order to induce PI3K activation, we treated the SKOV-3 cells with a low dose of rapamycin (2 nM) [20]. In addition, we added increasing concentrations of MRIA9 (Figure 1F). The analysis of phosphorylation levels confirmed that MRIA9-driven SIK2 inhibition reduces SIK2 auto-phosphorylation in cells (SIK2-pS385), and most importantly, strongly decreases rapamycin-induced AKT phosphorylation at low inhibitor concentration to levels comparable to those found before AKT stimulation (Figure 1F). Taken together, our results demonstrate the high potency of MRIA9 on endogenous substrates and SIK2 auto-activation in cellular systems.

### 2.2. Inhibition of SIK2 Reduced the Mitotic Index and Interfered with Spindle Assembly

Due to the role of SIK2 in mitosis, we hypothesized that SIK2 itself is cell cycle-regulated. Expression studies along the cell cycle using a thymidine synchronization of SKOV-3 cells showed that the SIK2 protein level is indeed cell cycle-regulated. SIK2 expression showed two peaks, one in the G1 phase (0 h) (Figure S1A,B) and one in the G2 phase concomitant to the increase in Cyclin B1 expression (9 h) (Figure S1A,B). Interestingly, high levels of SIK2 were still observed during early phases of mitosis (9–12 h) before SIK2 levels decreased during the metaphase to anaphase transition as indicated by the reduction in Cyclin B1 levels (Figure S1B). Immunofluorescence analysis of SIK2 localization during mitosis confirmed its presence at the centrosomes from prophase to anaphase, colocalized with CEP250 (Figure S1C). These results suggested a key role of SIK2 in regulating mitosis, likely by controlling centrosome function and their ability to assemble the mitotic spindle.

**Figure 2:**
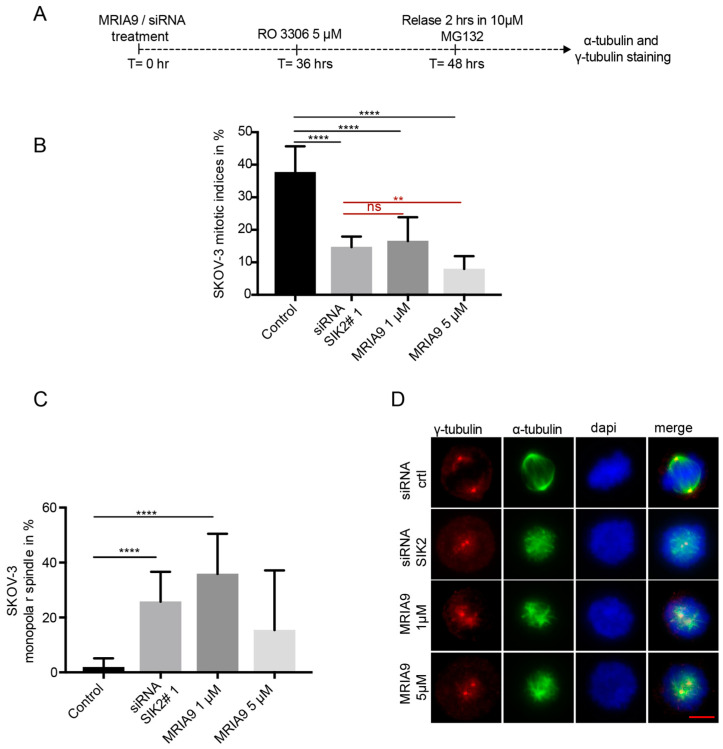
MRIA9 impedes G2-M transition and causes spindle assembly errors in SKOV-3 cells. (**A**) Scheme of the experimental procedure. SKOV-3 cells were treated with siRNA SIK2#1 or 1 µM and 5 µM MRIA9 for 36 h. Cells were then synchronized into the G2-phase using 5 µM RO3306 for 12 h. The synchronized cells were released for 2 h into mitosis in the presence of 10 µM MG132 to prevent cells from exiting mitosis. SKOV-3 cells were fixed and stained for IF using α-tubulin, γ-tubulin, and DAPI. (**B**) Mitotic indices in the different treatments were scored by microscopy. The results are represented as mean ± SD and statistically analyzed (**** *p* < 0.001, ** *p* < 0.01, ns: no significant). (**C**) The spindle phenotypes following the treatments were analyzed. Frequencies of cells with a monopolar spindle. The results are presented as mean ± SD and statistically analyzed (**** *p* < 0.001). (**D**) Representatives of SKOV-3 cells showing monopolar spindles after treatment with siRNA SIK2#1 and MRIA9. Scale bar = 10 µm.

As the inactivation of SIK2 by MRIA9 blocked the centrosome disjunction in late G2 synchronized SKOV-3 cells (Figure 1E), we asked whether inhibition of SIK2 affects mitotic entry and whether SIK2 loss of function interferes with mitotic spindle assembly. To investigate this, we treated SKOV-3 cells with siRNA SIK2#1 and two concentrations of MRIA9 (1 µM and 5 µM). Afterward, the cells were synchronized into the G2 phase using 5 µM RO3306, released for 2 h in 10 µM of the proteasome inhibitor MG132 to prevent cells from exiting mitosis (Figure 2A). Using IF, we calculated the mitotic indices of the different treatment groups. During the release time, knockdown of SIK2 or MRIA9-induced inhibition of SIK2 led to a significant and dose-dependent reduction in cells undergoing mitosis compared to control cells (Figure 2B). The mitotic indices decreased from 37.7% to 14.7%, 16.5%, and 8%, respectively, for controls, siRNA SIK2#1, MRIA9 1 µM, and MRIA9 5 µM treated cells, which indicated that abrogating SIK2 function, both by chemical inhibition of its catalytic activity as well as by genetic knockdown, interfered with the G2/M transition.

We further assessed the effects of SIK2 inhibition and knockdown on the spindle phenotypes and found that depletion or inhibition of SIK2 led to a significant reduction in normal and bipolar spindles compared to control cells (Figure S1D). Interestingly, in SKOV-3 cells, the main effect of the knockdown or the inhibition of SIK2 was the presence of mitotic spindles with almost unseparated or slightly separated centrosomes. We classified these spindles as monopolar spindles (Figure 2C,D). The increase in monopolar spindle rate caused by MRIA9 inhibition of SIK2 went from 1.9% (control) to 35.9% (1 µM MRIA9) and 15.5% (5 µM MRIA9), respectively (Figure 2C).

Similarly, inhibition of SIK2 in a second ovarian cancer cell line, A2780, also caused a dose-dependent and significant reduction in the mitotic indices compared to DMSO treated cells (DMSO, 34.2%; 1 µM MRIA9, 25.2%; 5 µM MRIA9, 18.5%) (Figure S2A,B). These data confirmed that inhibiting the catalytic activity of SIK2 interfered with mitotic entry. Interestingly, whereas 1 µM MRIA9 only showed a modest effect on the spindle bipolarity, 5 µM MRIA9 significantly reduced the frequency of bipolar spindles in mitotic A2780 cells compared to the DMSO control (Figure S2C). In contrast to SKOV-3 cells, in which monopolar spindles were the main consequence of inhibiting SIK2, inhibition of SIK2 in A2780 cells causes defects in spindle structure, and treatment of MRIA9 generated abnormal mitotic spindles (bipolar spindles with microtubules nucleation defects) associated with chromosome alignment failures (Figure S2D). The fraction of abnormal spindles increased from 8.6% (DMSO) to 21.2% and 22.1%, respectively, for cells treated with 1 µM MRIA9 and 5 µM MRIA9 (Figure S2E). Moreover, only the highest concentration of MRIA9 significantly increased the fraction of multipolar spindles compared to DMSO or with 1 µM treated cells (Figure S2F).

Altogether, the results showed that inhibition of the kinase activity of SIK2 reduced mitotic indices in two synchronized ovarian cancer cell lines. However, the consequences of SIK2 inhibition on centrosome function and spindle assembly appear to diverge between the two ovarian cell lines SKOV-3 and A2780, suggesting a cell-type specific response. Nevertheless, these phenotypic differences cannot be explained by variations in SIK2 protein levels in SKOV-3 and A2780, as both cell lines displayed similar SIK2 expression levels (Figure S2G).

### 2.3. Inhibition of SIK2 Altered the Positioning of the Mitotic Spindle

**Figure 3:**
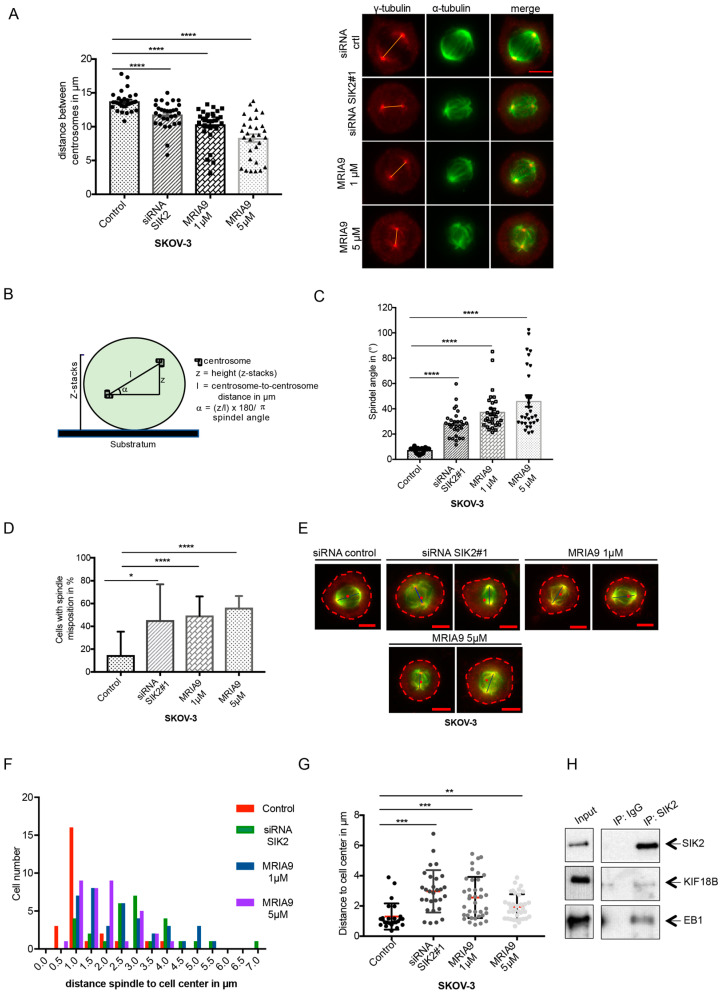
MRIA9-induced inhibition of SIK2 results in spindle position failures in SKOV-3 cells. (**A**) Inhibition of SIK2 using MRIA9 reduces the distance between the spindle poles. SKOV-3 cells were treated with siRNA SIK2#1, with 1 µM and 5 µM MRIA9. After 48 h, cells were immunostained with α-tubulin, γ-tubulin, and the pole-to-pole distance of prometa-metaphase cells was measured. (Left) The spindle lengths are presented as mean ± SD and statistically analyzed (**** *p* < 0.001, *n* = 30 cells). (Right) Representatives of SKOV-3 with reduced spindle length after depletion or inhibition of SIK2 using MRIA9. Scale bar = 10 µm. (**B**) Schematic representation of how the spindle axis is measured. The given formula is used to calculate the spindle angle. (**C**) The angles of the spindle axes in SKOV-3 cells treated with siRNA SIK2#1 or 1 µM and 5 µM MRIA9 were calculated and represented as mean ± SD and statistically analyzed (**** *p* < 0.001, *n* = 30 cells). (**D**) SIK2 depletion or inhibition using MRIA9 causes spindle mispositioning. The percentages of SKOV-3 cells displaying spindle mispositioning upon treatment with siRNA SIK2#1, 1 µM and 5 µM MRIA9 are represented as mean ± SD and statistically analyzed (**** *p* < 0.001, * *p* < 0.05, *n* = 30–38 cells). (**E**) Representatives of spindle position in control, siRNA SIK2#1, 1 µM MRIA9, and 5 µM MRIA9 treated cells. The cell edges are outlined, and the cell center is represented with a red dot. The blue line indicates the centrosome-to-centrosome distance. Scale bar = 10 µm. The spindle position of prometa-metaphase cells was evaluated by measuring the distance between the cell centroid and the center of the spindle (midpoint between the two centrosomes). (**F**) Histogram showing the offset distances assessed in the different treatment groups. (**G**) The distribution of the distances between cell center and spindle midpoint is represented as a scatter plot. The results were statistically analyzed (*** *p* < 0.001, ** *p* < 0.01, *n* = 25–38 cells). (**H**) The interaction between SIK2, EB1, and KIF18B was investigated in SKOV-3 cells by co-immunoprecipitation using SIK2 specific antibodies. The precipitates and 5% of the input were subjected to immunoblotting using the indicated antibodies.

Previous experiments demonstrated that SIK2 knockdown and inhibition prevented the complete separation of the centrosomes during early mitosis leading to spindle assembly failure. We next sought to measure the spindle length of SKOV-3 cells that were treated with siRNA SIK2#1 or MRIA9. Depletion of SIK2, as well as treatment with MRIA9, decreased the average pole-to-pole distance. Genetic knockdown reduced pole-to-pole distance from 13.7 µm to 11.8 µm, comparing RNAi-treated cells with the siRNA control. Inhibition of the catalytic activity showed even more drastic reductions to 10.3 µm and 8.3 µm, respectively for the 1 µM and 5 µM inhibitor-treated cells. These data strongly support that abrogation of SIK2 interferes with centrosome function by preventing the complete separation of the centrosomes in mitosis and reducing the centrosome ability to nucleate the spindle microtubules correctly (Figure 3A). Similar data were obtained from A2780 cells (Figure S3A).

We previously showed that MRIA9 impeded the appropriate centrosome positioning in interphase cells resulting in nuclear-centrosome uncoupling (NCU) (Figure 1C). This consequently prompts us to ask whether the cells that eventually enter mitosis with inhibited SIK2 may also present centrosome positioning errors, possibly leading to spindle orientation defects. To address this, we immunostained SKOV-3 cells that were treated with siRNA and MRIA9 for α- and γ-tubulin to visualize mitotic spindles and centrosomes, respectively. Using fluorescence microscopy, we performed z-stacks from pro-metaphase and metaphase cells of the different treatments. The centrosome-to-centrosome distance and the z-stacks range were used to calculate the angle between the mitotic spindle axis and the growth surface as described in earlier studies [21] (Figure 3B). While in control cells, the centrosomes were in similar z planes and almost parallel to the substratum; cells with depleted or inhibited SIK2 had spindle poles in substantially different z planes. We found that the RNAi-based knockdown and inhibition of SIK2 using MRIA9 significantly increased the average spindle axis angle from 7.3 degrees in controls to 28.2, 37.6, and 46.1 degrees in siRNA-treated cells and cells treated with 1 µM MRIA9, and 5 µM MRIA9, respectively. These data suggested a misalignment of the spindle in cells where SIK2 function was blocked (Figure 3C). Spindle misposition has also been confirmed in A2780 cells treated with MRIA9 (Figure S3B).

Additional experiments also confirmed errors in mitotic spindle positioning. During normal mitosis, the spindle localization matches the geometric center of the cell. However, SKOV-3 cells in which SIK2 was depleted or inhibited by MRIA9 frequently showed mitotic spindles that drift away from the cell center (Figure 3D–G). To quantify this, we determined the center of the spindle by calculating the distance between the two centrosomes. We determined the centroids of the cells, and then we assessed the offset distance between the spindle center and the centroid of the treated SKOV-3 cells. We observed that abrogating SIK2 activity or protein levels significantly raised the overall percentage of cells showing spindle misposition. In this experiment, control cells (DMSO) showed spindle misposition in 14.6% of all mitotic cells. In contrast, cells treated with siRNA (SIK2#1) or MRIA9 at 1 µM or 5 µM consistently displayed spindle misposition in 45%, 49%, and 56% of all mitotic cells, respectively (Figure 3D). The average offset distance separating the cell center and the spindle center went from 1.3 µm in control cells to 3 µm, 2.6 µm, and 2 µm, respectively in siRNA or inhibitor-treated cells (Figure 3E–G). Comparable results were obtained in the A2780 cell line. We observed a significant increase in the proportion of mitotic A2780 cells displaying centrosome mispositioning (Figure S3B–F). Thus, these results indicate that SIK2 activity seems to be required to establish the correct cell division plane by supporting the correct centrosome alignment and spindle position during mitosis.

Several lines of evidence indicated that the microtubule plus-end binding protein EB1 plays a critical role in defining the spindle position. Through mediating the interaction between microtubule ends, the cell cortex, and dynein motors, EB1 is involved in spindle assembly, dynamic, and positioning of the spindle [22,23,24]. Intriguingly, in our immunoprecipitation (IP) experiment using SIK2 antibodies, EB1 was found to precipitate with SIK2 in G2-synchronized SKOV-3 cells (Figure 3H). Furthermore, immunofluorescence staining of SIK2 and EB1 showed that both proteins colocalized at the spindle poles of mitotic SKOV-3 cells (Figure S3G). Interestingly, the SIK2-IP also revealed the presence of KIF18B, a kinesin-8 family member that complexed with SIK2 and EB1 in G2 synchronized cells (Figure 3H). Intriguingly, the association between KIF18B and SIK2 seemed to depend on the catalytic activity of SIK2 as treating unsynchronized or G2-cells with MRIA9 drastically reduced the association of SIK2 with KIF18B (Figure S3H). These data suggested that SIK2 might regulate KIF18B enzymatic activity through phosphorylation. This is an interesting finding as KIF18B is known to regulate astral microtubules (MTs), and its downregulation results in spindles with strong positioning defects [25]. Thus, our data suggest that SIK2 might promote centrosome alignment and spindle orientation during mitosis, likely through its interaction with EB1 and KIF18B.

### 2.4. Long-Term Inhibition of SIK2 Catalytic Activity Enhances Chromosomal Instability

**Figure 4:**
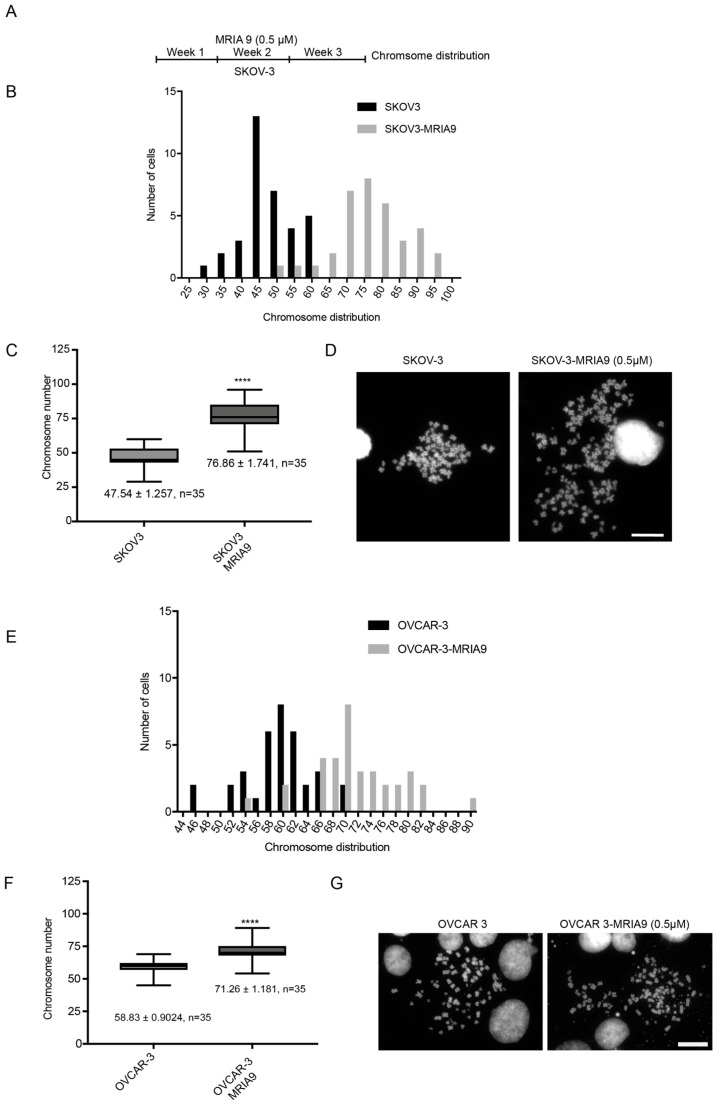
MRIA9-dependent inhibition of SIK2 increases chromosomal instabilities in ovarian cancer cells. (**A**) Scheme of the treatment schedule. SKOV-3 and OVCAR-3 cells were treated for three weeks with 0.5 µM MRIA9. Metaphase spreads of untreated controls and MRIA9 treated cells were prepared at the end of the incubation, and the chromosomes were stained with Hoechst. (**B**) Histogram plot of the distribution of chromosome numbers in SKOV-3 controls and SKOV-3 cells treated with MRIA9. (**C**) Quantification of the number of chromosomes in SKOV-3 controls and after incubation with 0.5 µM MRIA9 for three weeks. (mean ± SD). The results were statistically analyzed. **** *p* < 0.001. (**D**) Representatives of metaphase chromosome spreads. Scale bar = 20 µm. (**E**) Histogram displaying the distribution of chromosome numbers in OVCAR-3 controls and OVCAR-3 cells treated with 0.5 µM MRIA9. (**F**) Quantification of the number of chromosomes in OVCAR-3 controls and after incubation with 0.5 µM MRIA9 for three weeks. (means ± SD). The results were statistically analyzed. **** *p* < 0.001. (**G**) Representatives of OVCAR-3 metaphase chromosome spreads. Scale bar = 20 µm.

Properly functioning centrosomes are essential for faithful chromosome segregation. Impeding their function results in incorrect mitotic progression and hampers the accurate transmission of the genomic material [26]. Based on the role of SIK2 in accurate spindle positioning, we hypothesized that SIK2 inhibition might lead to chromosomal instability in ovarian cancer cells. To test this hypothesis, we treated the ovarian cancer cell lines SKOV-3 and OVCAR-3 continuously for three weeks with a low dose of MRIA9 (0.5 µM) (Figure 4A). We prepared chromosomal spreads at the end of the treatment period and determined the number of chromosomes in MRIA9-treated cells compared to their respective untreated counterparts. In both ovarian cell lines, the long-term inactivation of SIK2 using MRIA9 increased the whole chromosome number. SIK2 inactivation in SKOV-3 raised the mean chromosome number from 47.54 to 76.86, while in OVCAR-3, the number of chromosomes increased from 58.83 to 71.26, respectively (Figure 4B–G), demonstrating that inhibition of SIK2 activity promoted chromosomal instability in ovarian cancer.

### 2.5. Long-Term Inhibition of SIK2 Reduced 2D-Colony Forming Ability and Sensitized Ovarian Cancer Cells to Paclitaxel Treatment

**Figure 5:**
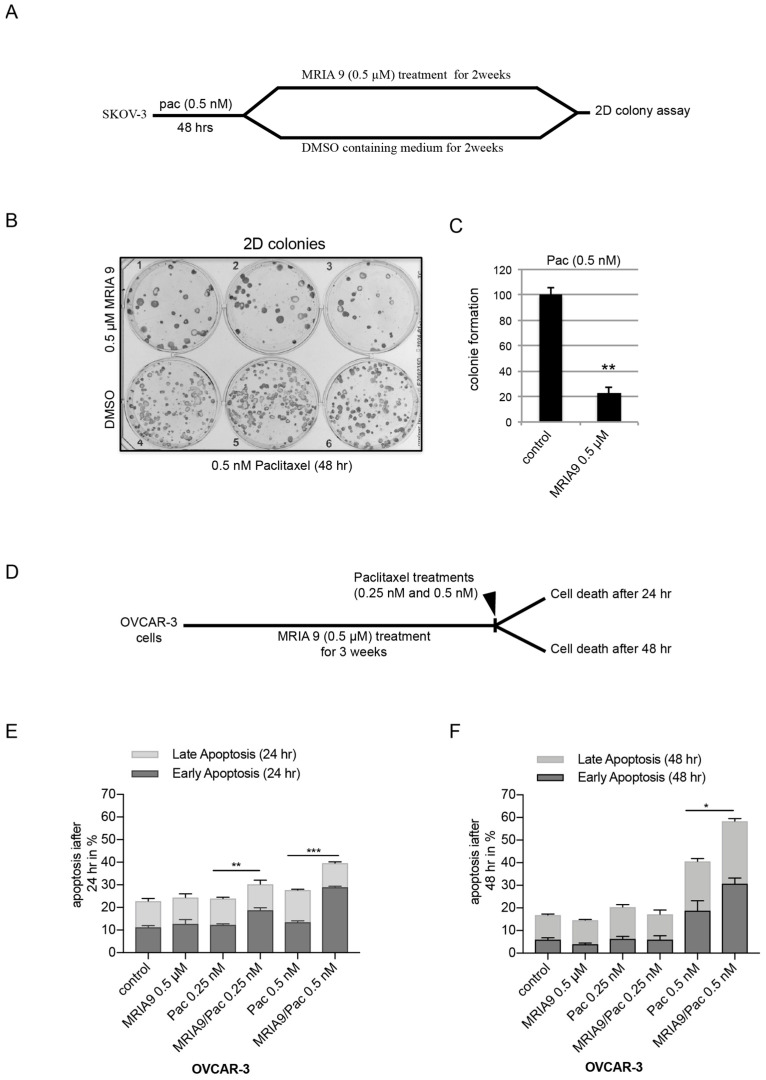
MRIA9-induced long-term inhibition of SIK2 decreases survival and sensitizes ovarian cancer cells to paclitaxel treatment. (**A**) Scheme of the experimental procedure. SKOV-3 cells were first incubated with 0.5 nM Pac for 48 h. On day two, cells were washed and incubated with and without 0.5 µM MRIA9 containing medium for two weeks. (**B**) SKOV-3 cells grown in colonies were subjected to Coomassie blue staining. (**C**) The number of colonies was determined and represented as a bar graph. (**D**) Scheme of the experimental procedure. OVCAR-3 cells were first treated for three weeks with 0.5 µM MRIA9. Subsequently, 0.25 nM and 0.5 nM Pac were added to the cultures. Cell death was then assessed after 24 h (**E**) and 48 h (**F**) using Annexin V/AAD. The results are presented as mean ± SD. The total apoptosis (early and late) was used for statistical analysis. *** *p* < 0.001, ** *p* < 0.01, * *p* < 0.05.

We studied the effects of the SIK2 long-term inhibition on the ability of ovarian cancer cells to survive and form colonies after paclitaxel treatment. SKOV-3 cells were first treated with a low dose of paclitaxel (0.5 nM) followed by treatment with 0.5 µM MRIA9 or DMSO containing medium for two weeks (Figure 5A). The inhibition of SIK2 combined with paclitaxel significantly reduced the ability of SKOV-3 cells to form colonies (100 colonies in the combination Pac/DMSO vs. 22 colonies for Pac/MRIA9 (Figure 5B,C)).

In a second long-term experiment, we incubated OVCAR-3 cells for three weeks with 0.5 µM MRIA9 and subsequently added to the cultures two low doses of paclitaxel (0.25 nM, or 0.5 nM). We then measured the induction of apoptosis upon paclitaxel addition using Annexin V/7-AAD assay (Figure 5D). After 24 h, while paclitaxel and MRIA9 as single agents did not lead to cell death (Figure 5E), the combinations of both agents significantly induced apoptosis. Notably, the highest paclitaxel concentration (0.5 nM) combined with SIK2 inhibition showed the highest apoptosis induction compared to their respective single treatments (27% in 0.5 nM Pac vs. 40% for the combination Pac/MRIA9) (Figure 5E). However, after 48 h, only the combination of MRIA9 with the highest paclitaxel concentration (0.5 nM) still significantly increased apoptosis compared to the single paclitaxel treatment (40% in 0.5 nM Pac vs. 58% for the combination Pac/MRIA9) (Figure 5F).

Together, diverse long-term treatment experiments showed that the cytotoxicity of paclitaxel can be significantly enhanced by simultaneous inhibition of SIK2. The combination of both agents also reduced colony formation of ovarian cancer cells.

### 2.6. The Combination of Inhibition of SIK2 with Paclitaxel Increases Apoptosis in Ovarian Cancer Cells 3D-Spheroids

**Figure 6:**
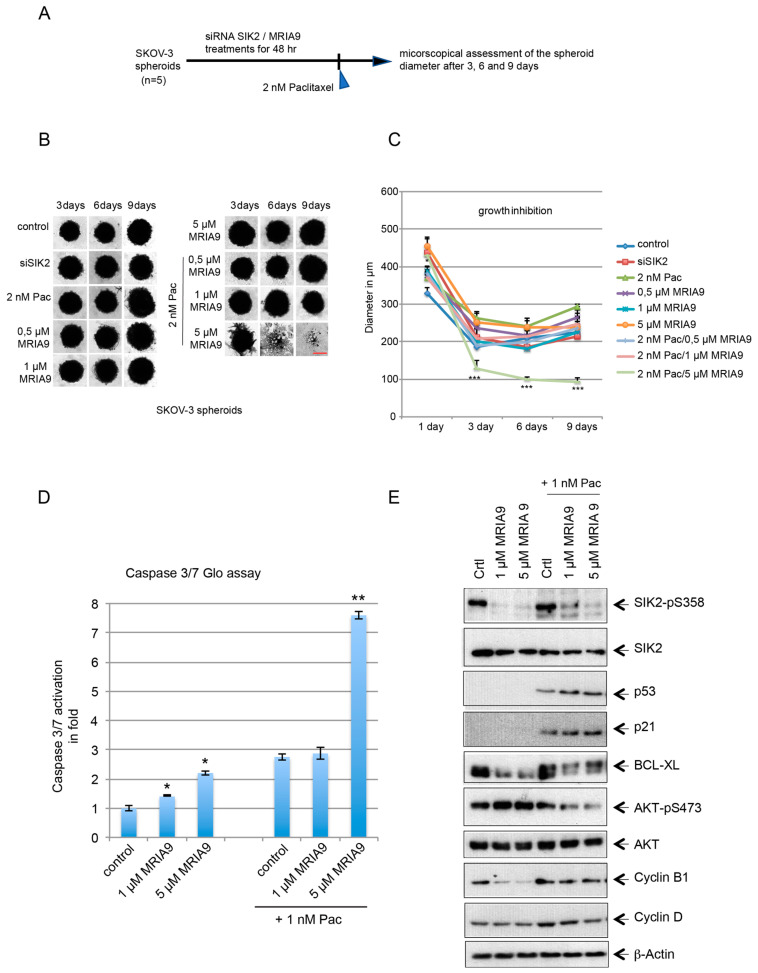
The combination Pac/MRIA9 reduces the growth of SKOV-3 spheroids. (**A**) 3D-spheroids grown from SKOV-3 cells were either SIK2 depleted or treated with 1 µM and 5 µM MRIA9 for 48h. Subsequently, 2 nM Pac was added to the spheroid cultures. The assessment of the spheroid growth was monitored using microscopy at the indicated time points, and the spheroid size measurements were performed using Image J. (**B**) Representative images of SKOV-3 spheroids that underwent single paclitaxel, MRIA9 treatments, or the combination Pac/MRIA9 between day 3 and 9. Scale bar = 150 µm. (**C**) Growth kinetics of the differently treated SKOV-3 spheroids represented in spheroid diameter over nine days. *** *p* < 0.001 (*n* = 5). (**D**) Caspase 3/7 activity measured in cell lysates of SKOV-3 cells incubated with single 1 µM and 5 µM MRIA9 or the combination Pac/MRIA9 for 48 h. The results are presented as mean fold ± SD and statistically analyzed. ** *p* < 0.01, * *p* < 0.05. (**E**) The lysates were prepared for Western blot analysis with the indicated antibodies.

3-dimensional spheroids are arguably one of the cell culture systems that mimic the native tumor environment more closely than a 2D cell cultures. We established SKOV-3 and OVCAR-3 3D-spheroids as described previously [27]. First, we treated the SKOV-3 spheroids with siRNA SIK2#1 or with increasing concentrations of MRIA9 (0.5, 1, and 5 µM) as a single treatment or in combination with 2 nM paclitaxel (Figure 6A). Paclitaxel and the dose-dependent MRIA9 single treatments showed no effect on the diameter of the spheroids compared to untreated cells (Figure 6B,C). The most substantial combinatorial effect was achieved after combining 2 nM Pac with 5 µM MRIA9. Already on day three we observed a significant reduction in the spheroid diameter compared to single MRIA9 and single paclitaxel treatment (Figure 6B,C). This decrease in spheroid volumes after the combinatorial treatment 5 µM MRIA9/Pac continued until day nine, where the mean volume decreased to 93 µm compared to 226 µm for controls and 292 µm for Pac 2 nM (Figure 6B,C). Further, we incubated SKOV-3 cells with 1 µM and 5 µM MRIA9, and we sequentially added 1 nM paclitaxel as a single or a combinatorial treatment. After 48 h, we measured caspase activation using a Casp3/7 Glo assay. Compared to untreated cells, the treatment with 1 µM and 5 µM MRIA9 significantly increased apoptosis in SKOV-3 cells as indicated by the activation of caspases (1.5 folds and 2.2 folds, respectively for 1 µM and 5 µM MRIA9 compared to control cells) (Figure 6D). However, only the highest combinatorial treatment, 1 nM Pac with 5 µM MRIA9, triggered the most consistent caspase activation compared to single paclitaxel treatment or even to the combination 1 nM Pac/1 µM MRIA9 (respectively 2.8 folds, 2.9 folds vs. 7.6 folds) (Figure 6D). Western blot analysis of the same treated cells confirmed the MRIA9 dose-dependent inactivation of SIK2 (reduction of pS385 signal) (Figure 6E). Moreover, the combination Pac/MRIA9 strongly decreased the phosphorylation of AKT (AKT-pS473), increased P53 and P21 expression, and reduced the anti-apoptotic protein BCL-xL compared to single paclitaxel treatment. These results suggested that the Pac/MRIA9 combination triggered a cell cycle arrest and enhanced apoptosis in SKOV-3 cells.

We performed the same sequential treatment in OVCAR-3 as a second ovarian cancer cell line. We treated the OVCAR-3 spheroids with siRNA SIK2#1 or two concentrations of MRIA9 (0.5 µM and 1 µM) for 48 h. Subsequently, we added 2 nM paclitaxel and incubated the cultures for several days (Figure S4A). We used spheroid size measurements to evaluate the efficacy of single and combinatorial treatments [28]. Over an incubation time of nine days, 2 nM paclitaxel or 0.5 µM MRIA9 alone did not affect spheroid growth, and the respective spheroid sizes at the end of the incubation time were similar to control spheroids (Figure S4B,C). Interestingly, SIK2 knockdown or inhibition using 1 µM MRIA9 significantly reduced the spheroid growth after nine days compared to control cells. The spheroid diameter decreased from 430 µm to 338 µm and 295 µm, respectively for controls, siRNA SIK2#1, and 1 µM MRIA9 treatments (Figure S4B,C). The double combination, particularly the combination 2 nM Pac/1 µM MRIA9, resulted in the most pronounced inhibition of spheroid growth compared to single and double combination of paclitaxel with 0.5 µM MRIA9. After 9 days of incubation, the spheroid mean size decreased significantly to 91 µm for the combination 2 nM Pac/1 µM MRIA9 compared to 430 µm, 427 µm, 295 µm, and 301 µm, respectively for the controls, 2 nM Pac, 1 µM MRIA9, and the combination 2 nM Pac/0.5 µM MRIA9 (Figure S4B,C).

### 2.7. MRIA9-Induced Inhibition of SIK2 Improves the Paclitaxel Response and Increased Cell Death in Ovarian Patient derived Spheroid Cultures

**Figure 7:**
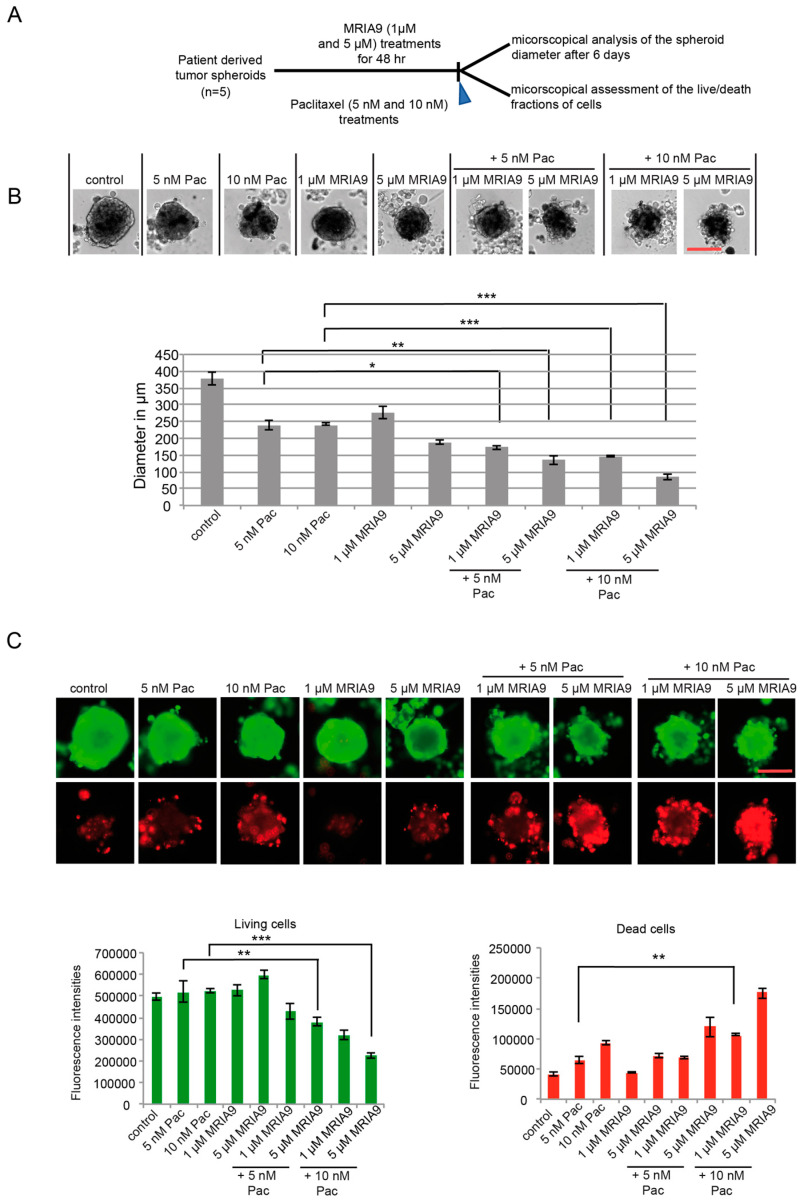
The combination Pac/MRIA9 blocks the growth and strongly enhances apoptosis-dependent death of patient derived ovarian tumor cells. (**A**) Scheme of the experimental procedure. 3D spheroids grown from tumor cells of primary patients were treated with 1 µM and 5 µM MRIA9 for 48 h. 5 nM and 10 nM paclitaxel were subsequently added to the spheroid cultures for 6 days. At the end of the incubation time, the spheroid diameters were assessed microscopically, and the live/dead fractions were quantified using immunofluorescence. (**B**) (Upper) Representative images of the patient derived spheroids that underwent single Pac and MRIA9 treatments or the combination Pac/MRIA9 after six days. Scale bar = 150 µm. (Lower) Growth inhibition after six days represented by the spheroid diameters is displayed as mean ± SD (*** *p* < 0.001, ** *p* < 0.01, * *p* < 0.05 (*n* = 5). (**C**) Patient derived spheroids were stained for immunofluorescence, and the live/ dead fractions were quantified. (Upper) Representative images of the patient derived spheroids that underwent the different treatments Scale bar = 150 µm. (Lower left) The fluorescence intensities of the living fraction in the different treatment groups were quantified and represented as mean ± SD. *** *p* < 0.001, ** *p* < 0.01. (Lower right) The fluorescence intensities of the dead fraction in the different treatment groups were quantified and represented as mean ± SD. ** *p* < 0.01.

Next, we asked whether blocking SIK2 activity using MRIA9 enhances the paclitaxel response of 3D-primary cancer cell cultures derived from patients diagnosed with ovarian cancer. Similar to the previous experiments with the ovarian cancer cell lines, we tested the combination 5 nM and 10 nM Pac with 1 µM and 5 µM MRIA9 using the same treatment schedule (Figure 7A). Interestingly, both MRIA9 concentrations combined with 5 nM or 10 nM paclitaxel reduced the sizes of the spheroids significantly compared to single paclitaxel or single MRIA9 treatments after 6 days (Figure 7B). However, the highest combination 10 nM Pac with 5 µM MRIA9, achieved the most prominent spheroid diameter reduction. The size of the spheroids decreased from 240 µm to 80 µm, respectively, for 10 nM paclitaxel vs. the combination 10 nM Pac/5 µM MRIA9 (Figure 7B). Live/death assays performed in the differently treated spheroid cultures after six days corroborated the previous result. The combination 10 nM Pac/5 µM MRIA9 resulted in the most robust increase in the death fraction of cells as opposed to the single paclitaxel treatment (Figure 7C). The results of this assay could be confirmed using a second 3D-spheroid culture originating from a second ovarian cancer patient (Figure S5A–C). Together, the results of the cell line-based and the patient tumor-derived 3D-spheroid assays support the clinical significance of the combination SIK2 inhibition with paclitaxel. More importantly, the data demonstrated the excellent potency of MRIA9 in targeting SIK2 and using patient derived primary cells, making it a promising candidate for more translational studies into ovarian cancer.

## 3. Discussion

SIK2 is a serine/threonine kinase and a member of the AMPK family. Its activity has been linked with many biological processes such as gluconeogenesis, insulin signaling, neuronal survival, and melanogenesis [11,29,30,31,32]. Furthermore, SIK2 has been identified as a mitotic regulator by influencing the centrosome function, and several studies described an oncogenic role of SIK2 in many cancer types [8,9,10,11,12]. Particularly in ovarian cancer, the overexpression of SIK2 in the adipocyte-rich omentum drives metastasis by promoting fatty acid oxidation and activating the PI3K pathway through direct phosphorylation [9]. Furthermore, SIK2 overexpression has been described in almost 30% of ovarian cancer patients and was associated with a poor prognosis [9,10]. All these findings suggest that the inhibition of SIK2 may present a novel and promising therapeutic avenue for ovarian cancer treatment, hence the need to develop new potent and selective SIK2 inhibitors. Due to the lack of crystal structure of SIK proteins, we based our development of the new SIK inhibitor on the scaffold of the pyrido[2,3-d]pyrimidin-7-one PAK inhibitor G-5555 [33]. By exploiting the differences in the back-pocket region of the SIK protein family, we developed a G-5555 derivative, MRIA9, that potently and selectively inhibits SIK2 [15]. We observed that the specific MRIA9-induced inhibition of SIK2 reduced its enzymatic activity in ovarian cancer cells monitored by measuring the autophosphorylation on Ser385. It was an attractive strategy to compare the activity of a selective small-molecule SIK2 inhibitor with the effects of SIK2 knockdown in causing nuclear centrosome uncoupling, preventing centrosome disjunction during late G2, and reducing the intracellular phosphorylation of AKT (pS473) after the PI3K activating treatment with rapamycin. These various assays confirmed the specificity and potency of MRIA9 in blocking the activity of SIK2.

Previous siRNA screen identified SIK2 as a regulator of G2/M progression in ovarian cancer [12]. Accordingly, treating synchronized SKOV-3 and A2780 cells with MRIA9 impeded the G2/M entry, increased spindle assembly failure, which reflects, most likely, the effect of MRIA9-dependent SIK2 inhibition on the centrosome function.

However, the finding that SIK2 inhibition using MRIA9 resulted in spindle misposition during early mitosis was very intriguing because proper spindle orientation and positioning are critical in ensuring symmetric division during mitosis and essential to secure the genomic stability in the cells. In this context, astral microtubules anchor the mitotic spindle to the cell cortex, setting the spindle orientation [34,35]. End binding protein 1 (EB1) localizes at the microtubule plus-end and regulates the dynamics of astral microtubules by recruiting other plus-end tracking proteins (+TIPs) [36]. In human cells, EB1 controls the length of astral MTs during mitosis via direct interaction and recruitment of the MT-depolymerase KIF18B to MT plus-ends. Moreover, blocking this association or the loss of KIF18B increases the length of astral MT and interferes with the spindle centering during mitosis [25,37]. Our SIK2 co-immunoprecipitation assay using specific antibodies showed the existence of both astral MT regulators, EB1 and KIF18B, with SIK2 in the same complex. Moreover, an SIK2 kinase activity-dependent association with KIF18B could be demonstrated using co-immunoprecipitation. Indeed, MRIA9-dependent inhibition of SIK2 strongly reduced its interaction with KIF18B, suggesting that SIK2 might be involved in regulating astral MT through modulating KIF18B depolymerase activity. Together, the co-IP results, when combined with the fact that blocking SIK2 activity interferes with the centrosome function (blocking centrosome disjunction during late G2) and induces spindle nucleation defects (shorter spindle, monopolar and aberrant spindles), indicate a role of SIK2 in establishing the cell division plane and ensuring the correct spindle position. However, the detailed mechanisms of how SIK2 promotes the spindle orientation, whether through phosphorylation and activation of KIF18B depolymerase activity or by promoting the recruitment and association of EB1 and KIF18B at the centrosome, remains to be elucidated and requires further mechanistic studies.

Malfunctioning centrosomes and spindle positioning defects may result in erroneous mitosis, leading to chromosome congression failure, impairing the genetic material’s transmission, thus facilitating genomic instability [26,38]. Consistent with this, we found that the long-term inhibition of SIK2 using MRIA9 significantly enhanced the chromosome number of SKOV-3 and OVCAR-3 cells, suggesting that SIK2 may play a critical role in maintaining genomic stability in ovarian cancers.

The potential of our new SIK2 inhibitor, MRIA9, was also demonstrated in 3D- spheroid assays of two ovarian cancer cell lines, SKOV-3 and OVCAR-3, and in primary ovarian cancer cells originating from different patients. Our experiment showed that MRIA9-dependent inhibition of SIK2 enhanced the paclitaxel sensitivity in all experimental models of ovarian cancer. Pac/MRIA9 combination significantly blocked the growth and reduced the spheroid volumes in addition to enhancing apoptosis-dependent cell death compared to the respective single treatments.

Together, our results demonstrate the efficiency and potency of the newly designed SIK2 inhibitor MRIA9 and confirm the benefit of combining SIK2 inhibition with a taxane-based treatment as a new tumor-suppressing strategy in this highly lethal gynecological disease.

## 4. Material and Methods

### 4.1. Cell Culture

The ovarian carcinoma cell lines OVCAR-3, SKOV-3, and A2780 were cultured in RPMI 1640 (Gibco, Thermo Fisher Scientific, Waltham, MA, USA) and in McCoy’s 5a (Gibco), respectively, both containing 10% FCS (Gibco) and 1% Penicillin/Streptomycin (Sigma-Aldrich, St. Louis, MO, USA). Primary cells were isolated from ovarian cancer tissues using the tumor dissociation kit (Max Miltenyi 130-095-929, Bergisch Gladbach, Germany) together with the tumor cell isolation kit (Max Miltenyi 130-108-339) following the manufacturer’s instructions.

### 4.2. Patients and Samples (Primary Cell Culture)

This study was conducted according to the “REporting recommendations for tumor MARKer prognostic studies” [39]. To establish primary, patient derived ovarian cancer cell cultures, we analyzed samples from patients undergoing surgical resection between January 2015 and March 2018 at the Department of Gynecology of the Goethe University Hospital in Frankfurt am Main, Germany. For the samples with validated diagnosis, sufficient archival material for immunohistochemical analysis was available. The Local Research Ethics Committees approved studies of human tissue, and samples were processed anonymously.

### 4.3. Colony Formation Assay

2000 cells were seeded in 6-well plates. Cells were treated with paclitaxel for 48 h, washed, and incubated in a fresh medium for two weeks. In the case of the combinatorial treatment PAC/MRIA9. The cells were first treated for 48 h with paclitaxel and then incubated in MRIA9 containing medium for two weeks. Colonies were fixed using 70% EtOH and stained with Coomassie Brilliant Blue. The numbers of grown colonies were counted, and images were taken using AxioObserver Z1 microscope (Zeiss, Göttingen, Germany) as well as the ChemiDoc MP system (BioRad, Hercules, CA, USA).

### 4.4. Three-Dimensional (3D) Cultures

A cell suspension of 1000 cells/50 μL was prepared and pipetted from the topside into a 96-well Perfect 3D Hanging Drop plate (BioTrend, Köln, Germany). Plates were incubated at 37 °C for one day until hanging drops had developed. The 3D culture was harvested on a 96-well plate covered with 1% agarose by low-spin centrifugation. Treatment of cells with MRIA9, paclitaxel was performed as indicated. Cells were stained with the LIVE/DEAD viability/cytotoxicity kit (Molecular Probes/Thermofisher) for 30 min and inspected using a fluorescence microscope. While the polyanionic dye calcein is retained in live cells, producing an intense uniform green fluorescence, EthD-1 enters cells with damaged membranes, thereby producing a bright red fluorescence upon binding to nucleic acids in dead cells. The ratios of viable/dead cells were calculated with the software ImageJ Fiji.

### 4.5. Chromosome Spreads

After incubation for three weeks with MRIA9, cells were treated overnight with 3.3 μM Nocodazole. The next day, cells were harvested by mitotic shake-off and hypotonically swollen in 0.75 mM KCL for 20 min at 37 °C. Cells were fixed with freshly made Carnoy’s solution (75% methanol, 25% acetic acid), and the fixative was changed several times. For spreading, cells in Carnoy’s solution were dropped onto prechilled glass slides. Slides were dried at room temperature for 24 h and stained with DAPI. Chromosome number per cell and condition was counted using an AxioObserver Z1 microscope with an HCX PL APO CS 63.0x1.4 oil UV objective (Zeiss, Göttingen). The graphic representation of the results was done using GraphPad Prism software.

### 4.6. Cell Cycle and Apoptosis Assay

For cell cycle analysis, cells were harvested, washed, fixed with 70% EtOH, and stained as described [40]. Cell cycle quantification was performed using FACS Calibur and Cellquest Pro software (both BD Biosciences, Franklin Lakes, NJ, USA). The activity of Caspase-3/7 was determined using the Caspase-Glo 3/7 Assay (Promega, Madison, WI, USA). Twenty microliters of substrate per well were applied, and after 30 min shaking at room temperature in the dark, luminescence was detected (Victor X4, Perkin Elmer, Waltham, MA, USA). The analysis of data was done online by Network analyst and GraphPad Prism.

### 4.7. Synchronization

To synchronize the cells into the G1/S boundary, cells were incubated with 2 mM thymidine (Sigma; 16 h), washed extensively with PBS, and released in fresh medium (8 h), followed by a second thymidine block (16 h). To synchronize the cells into the G2-phase, cells were incubated with the CDK1 inhibitor RO3306 (5 µM) for 12 h. To synchronize SKOV-3 and A2780 into metaphase, cells were first treated with 5 µM RO2206 for 12 h. Subsequently, the cells were washed and released in a fresh medium containing the proteasome inhibitor MG132 (10 µM) for 2 h.

### 4.8. Cold Kinase Assay In Vitro

GST-tagged SIK2 was incubated with 100 μM nonradioactive ATP at 30 °C for 30 min. The reaction was stopped by adding sample buffer and boiling for 5 min. Equal volumes of each reaction were loaded onto 10% SDS-PAGE gel and separated. The Western blots were probed for the indicated antibodies.

### 4.9. Western Blot Analysis, Antibodies and Chemicals

Cell lysis and Western blot analysis were performed as described previously [40], using the following antibodies: Rabbit polyclonal anti-SIK2 (Cell Signaling, Danvers, MA, USA), rabbit polyclonal anti-SIK2-pS385 (Kinexus, Sydney, Australia), rabbit polyclonal anti-AKT (Cell Signaling), rabbit polyclonal anti-AKT-pS473 (Cell Signaling), mouse monoclonal anti-β-actin (Sigma-Aldrich), rabbit polyclonal-anti KIF18B (ThermoFisher scientific, Waltham, MA, USA), rabbit polyclonal anti-EB1 (Abcam, Cambridge, UK), mouse monoclonal anti-cyclin B1 (Santa Cruz Biotechnology, Dallas, TX, USA), mouse monoclonal anti-Histone 3-pS10 (ThermoFisher scientific), mouse monoclonal anti-PLK1 (Santa Cruz Biotechnology), mouse monoclonal anti-vinculin (Santa Cruz Biotech), mouse monoclonal anti-P53 (Santa Cruz Biotech), rabbit polyclonal anti-P21 (cell signaling), rabbit polyclonal anti-BCL-xL (Santa Cruz Biotechnology), rabbit polyclonal anti-Cyclin D (Santa Cruz Biotechnology).

Reagents were purchased from the following sources: Paclitaxel (T7402) Sigma-Aldrich, propidium iodide (440300250) Acros Organics (Fair Lawn, NJ, USA), RNase A (1007885) Qiagen (Hilden, Germany), PE Annexin V (556421) and 7AAD (21-68981E) BD Biosciences, Caspase-Glo^®^ 3/7 Assay system (P1781) Promega, RO3306 (S7747) Selleckchem (Houston, TX, USA).

### 4.10. Immunofluorescence Assays

Cells were grown on a glass coverslip, fixed and permeabilized with methanol (−20 °C), and washed with PBS before adding appropriate primary and secondary antibodies. Cells were stained with the following primary antibodies: fluorescein isothiocyanate (FITC)-conjugated anti-α-tubulin (Sigma-Aldrich), mouse anti-γ-tubulin (Sigma, Germany), rabbit anti-CEP250 (Proteintech, Rosemont, IL, USA), rabbit anti-Rootletin (Santa Cruz Biotech), rabbit anti-SIK2 (Cell signaling), mouse anti-SIK2 (Biolegend, San Diego, CA, USA), and rabbit anti-EB1 (Santa Cruz Biotech). The secondary antibodies: FITC-conjugated donkey anti-mouse, FITC-conjugated goat anti-rabbit, or Cy3 conjugated goat anti-mouse (Jackson Immunoresearch, West Grove, PA, USA). DNA was stained using DAPI (4′,6-diamidino-2-phenylindol-dihydrochloride) (Roche, Mannheim, Germany). Slides were examined using an Axio Imager 7.1 microscope (Zeiss, Göttingen, Germany), and images were taken using an Axio Cam MRm camera (Zeiss, Göttingen, Germany).

### 4.11. Statistical Analysis

All experiments were performed at least three times and displayed as mean and standard deviation or standard error of the mean. The statistical significance was assessed by Student’s *t*-test (two-tailed and paired) using Excel 2010 (Microsoft) as well as GraphPad Prism 7 (GraphPad, La Jolla, CA, USA). Significant differences (* *p* ≤ 0.05; ** *p* ≤ 0.01; *** *p* ≤ 0.001) are indicated in the figures with asterisks.

## 5. Conclusions

The current standard therapy of ovarian cancer is based on the combination of platinum-taxane primary chemotherapy. Although the clinical response rate reaches 70%, only 20% of patients with advanced disease can be cured. Hence, there is an urgent need to find combinatorial therapies that permit overcoming the paclitaxel-associated resistance in patients. Our study reports the effects of SIK2 inhibition in living cells using MRIA9, a new selective and potent small-molecule inhibitor. MRIA9 revealed a new role of SIK2 in promoting the establishment of an accurate cell division plane through orchestrating the centrosome alignment and spindle position during the cell division and thus suggesting a role of SIK2 in maintaining chromosomal stability in ovarian cancer. We also showed using 3D-spheroid models of ovarian cancer cells and patient derived material that MRIA9-induced inhibition of SIK2 enhanced the sensitivity of ovarian cancer to paclitaxel therapy. Hence, the data presented in this study indicate that MRIA9 might represent a novel therapeutical avenue for translational studies to overcome paclitaxel resistance in ovarian cancer.

## Data Availability

The data and materials that support the findings of this study are available within this article and from the corresponding authors upon reasonable request.

## Supplementary Materials

The following are available online at https://www.mdpi.com/article/10.3390/cancers13153658/s1: Figure S1. SIK2 is a centrosome kinase, and its expression is cell cycle-regulated. Figure S2. MRIA9-dependent inhibition of SIK2 reduces the mitotic index and causes abnormal spindles in A2780 cells. Figure S3. MRIA9-induced inhibition of SIK2 results in spindle position failures in A2780 cells. Figure S4. MRIA9-induced inhibition of SIK2 in combination with paclitaxel reduces the growth of OVCAR-3 3D-spheroids. Figure S5. The combination Pac/MRIA9 enhances cell death in patient-derived ovarian tumor cells.
