# Supplementary figures and images for: The Small-Molecule Inhibitor MRIA9 Reveals Novel Insights into the Cell Cycle Roles of SIK2 in Ovarian Cancer Cells
Source: Cancers (Basel). 2021 Jul 21;13(15):3658. doi: 10.3390/cancers13153658 (PMC8345098; doi:10.3390/cancers13153658)

Figure S1

A

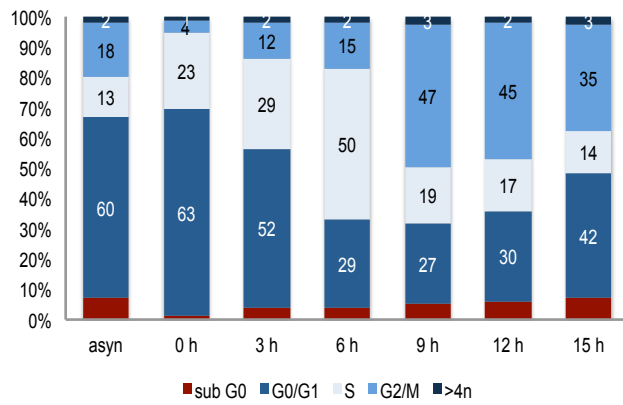

B

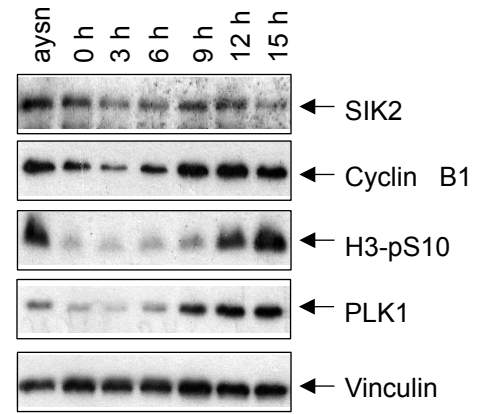

C

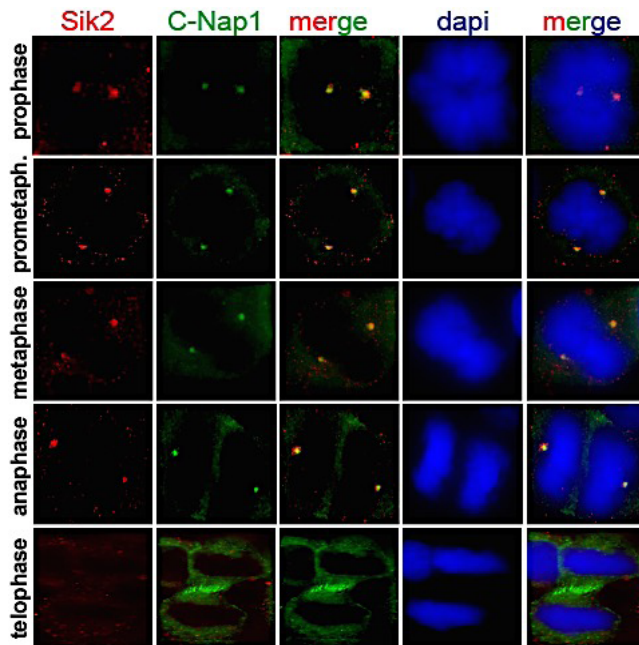

D

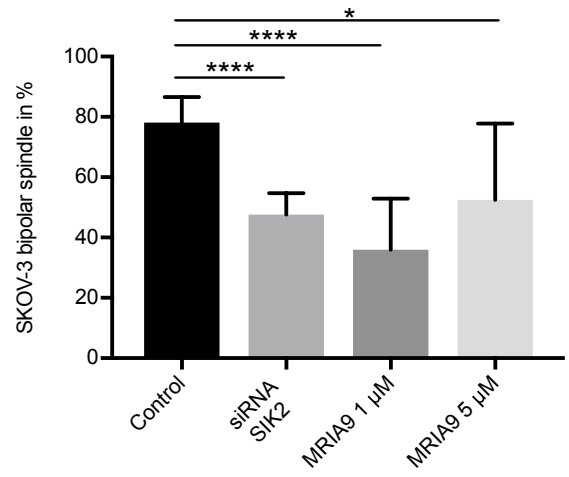

Supplement: Supplementary file 1 [file cancers-13-03658-s001.zip › Figure S1.pdf]

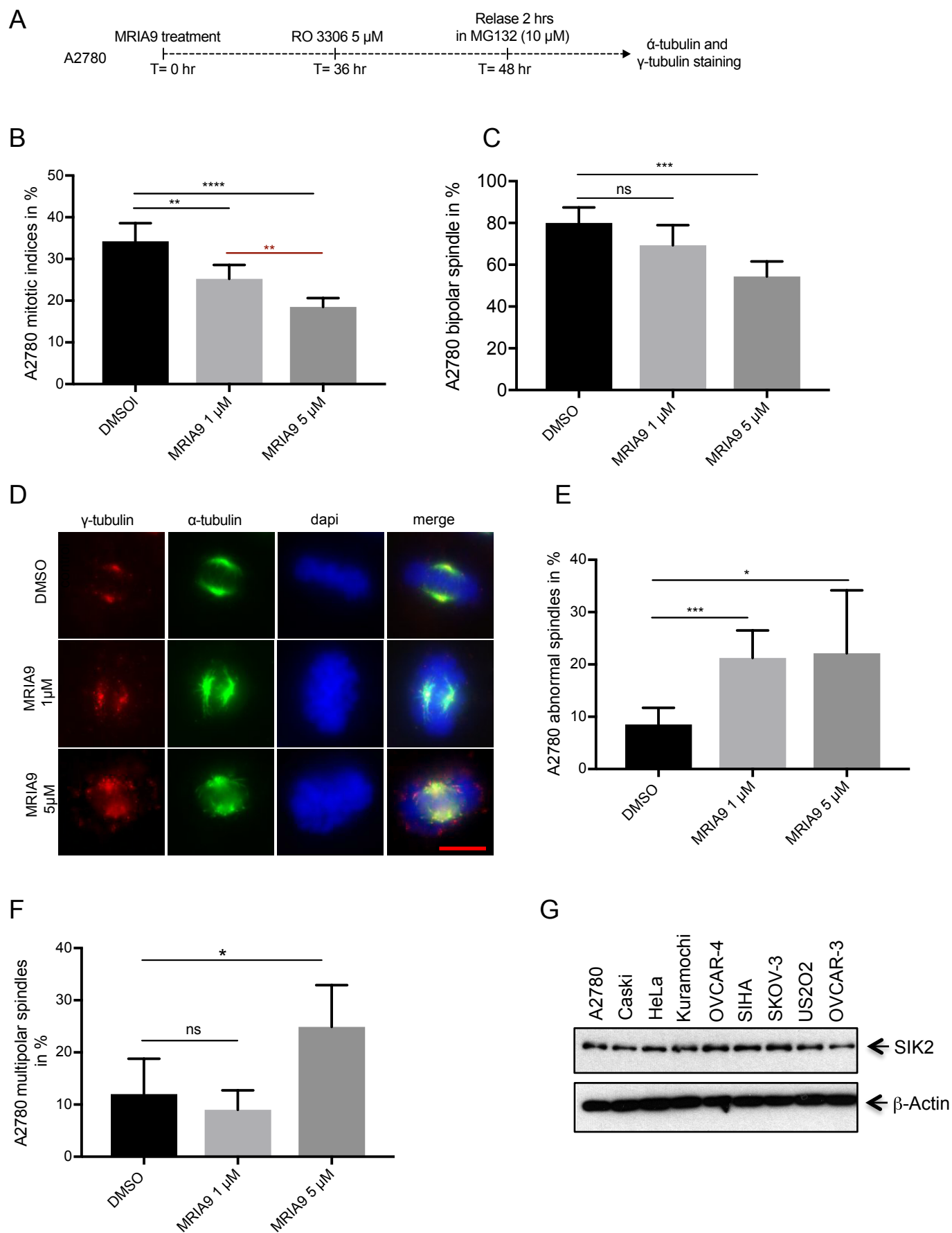

Supplement: Supplementary file 1 [file cancers-13-03658-s001.zip › Figure S2.pdf]

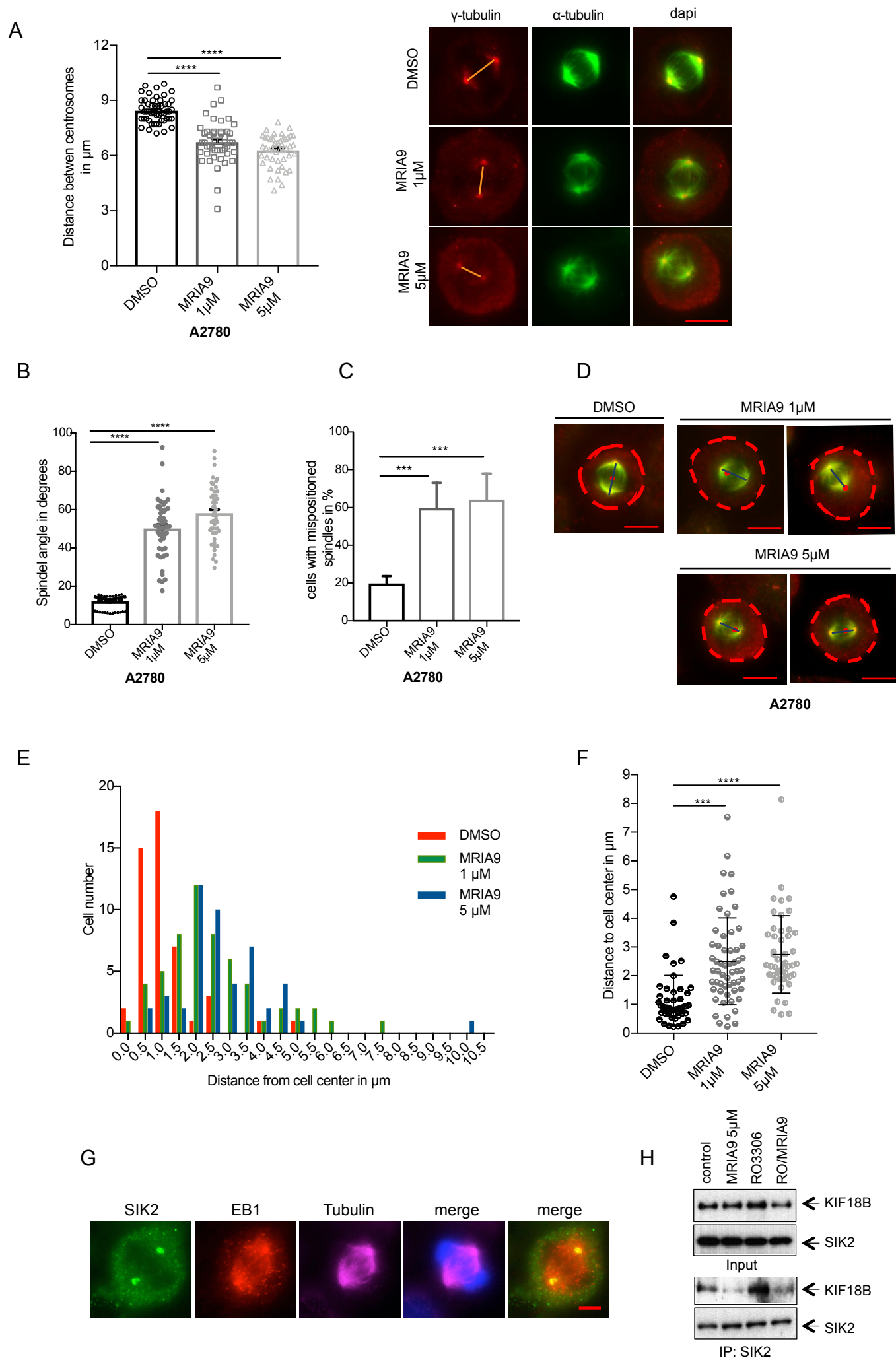

Supplement: Supplementary file 1 [file cancers-13-03658-s001.zip › Figure S3.pdf]

Figure S4

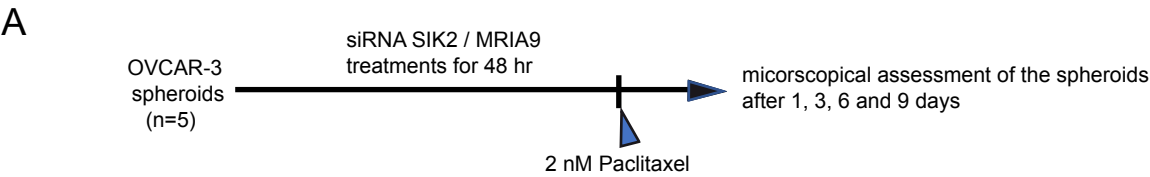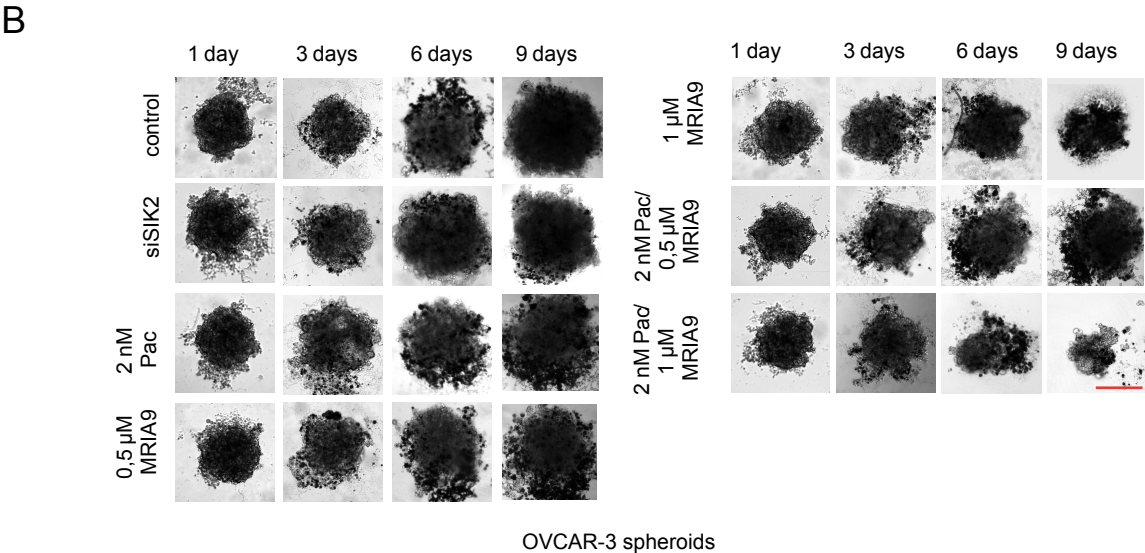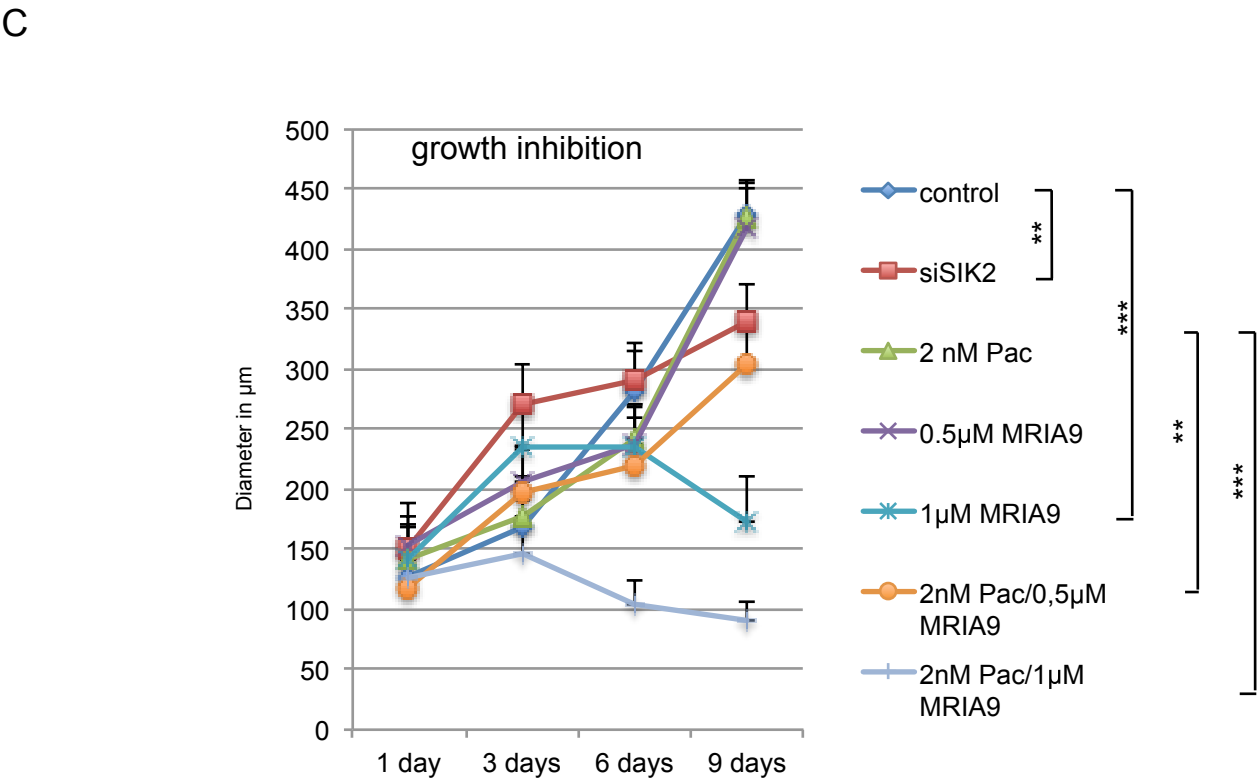

Supplement: Supplementary file 1 [file cancers-13-03658-s001.zip › Figure S4.pdf]

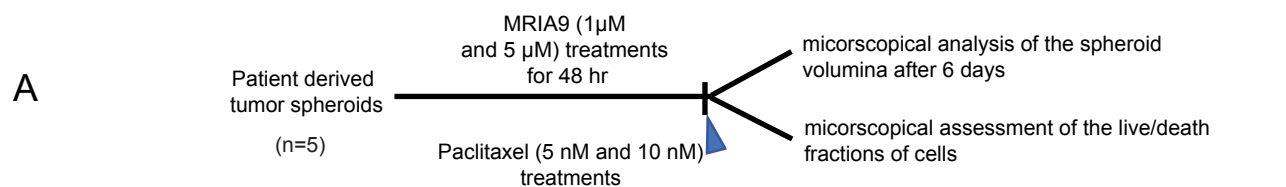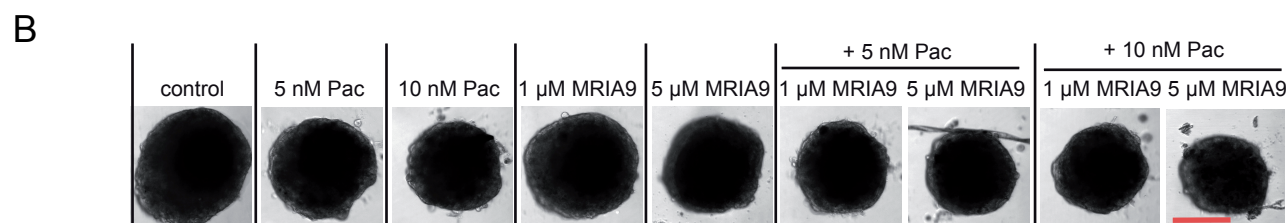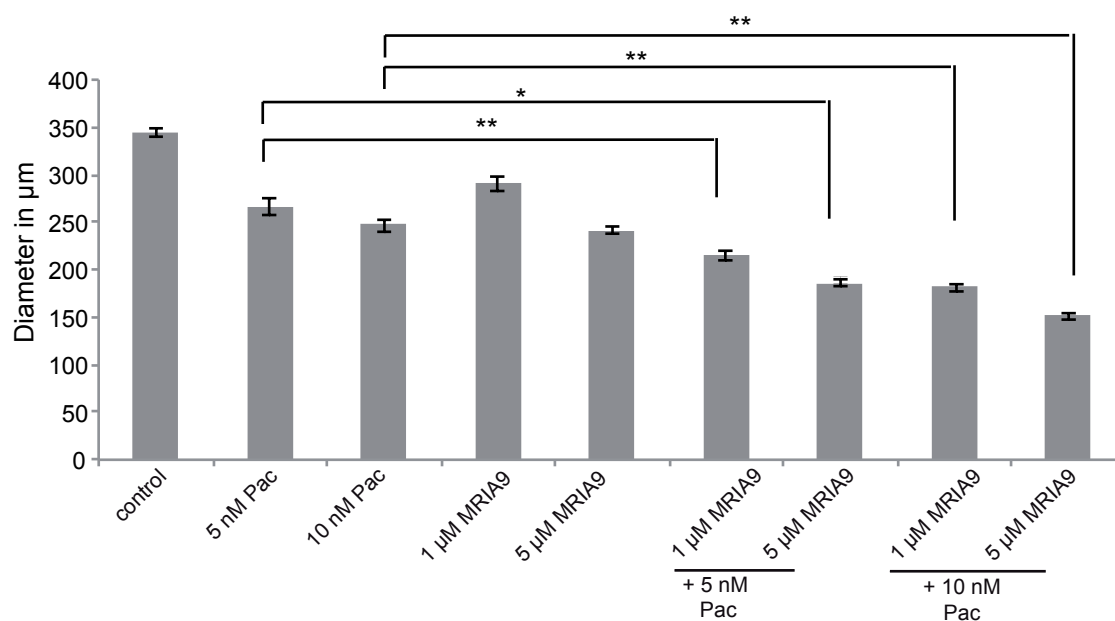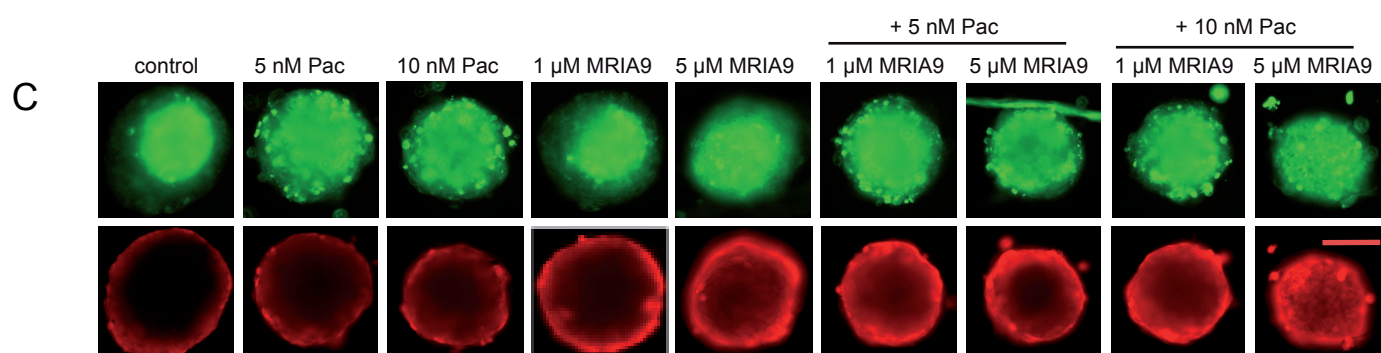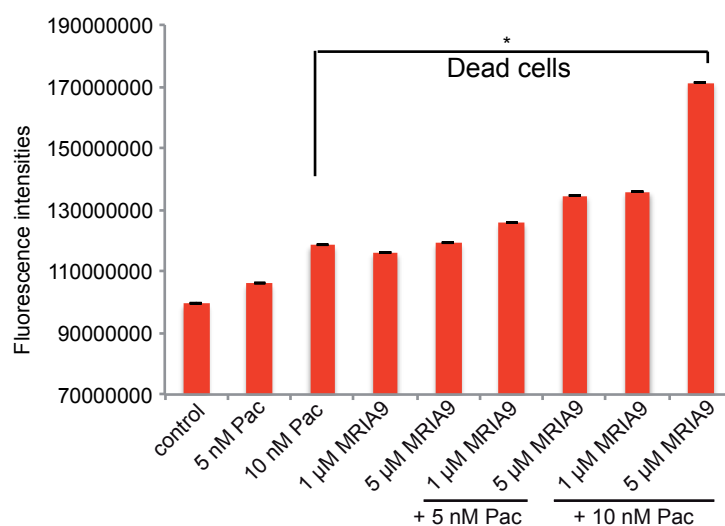

Supplement: Supplementary file 1 [file cancers-13-03658-s001.zip › Figure S5.pdf]
